# Transdermal Composite Microneedle Composed of Mesoporous Iron Oxide Nanoraspberry and PVA for Androgenetic Alopecia Treatment

**DOI:** 10.3390/polym12061392

**Published:** 2020-06-22

**Authors:** Jen-Hung Fang, Che-Hau Liu, Ru-Siou Hsu, Yin-Yu Chen, Wen-Hsuan Chiang, Hui-Min David Wang, Shang-Hsiu Hu

**Affiliations:** 1 Department of Biomedical Engineering and Environmental Sciences, National Tsing Hua University, Hsinchu 300, Taiwan; s102012803@m102.nthu.edu.tw (J.-H.F.); u9912052@gms.ndhu.edu.tw (C.-H.L.); hsu.ru.siou@gmail.com (R.-S.H.); anny99943@gmail.com (Y.-Y.C.); 2 Department of Chemical Engineering, National Chung Hsing University, Taichung 402, Taiwan; whchiang@dragon.nchu.edu.tw; 3 Graduate Institute of Biomedical Engineering, National Chung Hsing University, Taichung 402, Taiwan; davidw@dagon.nchu.edu.tw

**Keywords:** 3D printing process, mesoporous iron oxide, microneedles, minoxidil

## Abstract

The transdermal delivery of therapeutic agents amplifying a local concentration of active molecules have received considerable attention in wide biomedical applications, especially in vaccine development and medical beauty. Unlike oral or subcutaneous injections, this approach can not only avoid the loss of efficacy of oral drugs due to the liver’s first-pass effect but also reduce the risk of infection by subcutaneous injection. In this study, a magneto-responsive transdermal composite microneedle (MNs) with a mesoporous iron oxide nanoraspberry (MIO), that can improve the drug delivery efficiency, was fabricated by using a 3D printing-molding method. With loading of Minoxidil (Mx, a medication commonly used to slow the progression of hair loss and speed the process of hair regrowth), MNs can break the barrier of the stratum corneum through the puncture ability, and control the delivery dose for treating androgenetic alopecia (AGA). By 3D printing process, the sizes and morphologies of MNs is able to be, easily, architected. The MIOs were embedded into the tip of MNs which can deliver Mx as well as generate mild heating for hair growth, which is potentially attributed by the expansion of hair follicle and drug penetration. Compared to the mice without any treatments, the hair density of mice exhibited an 800% improvement after being treated by MNs with MF at 10-days post-treatment.

## 1. Introduction

For male baldness, this genetic disease occurs at age 30–60, which is estimated that over 50% of the general population suffer from hair loss [1]. Traditionally in the treatment of hereditary male baldness, the drug was mixed into the inactive component which can soften the stratum corneum, such as glycerol and propylene glycol, facilitating the active ingredient effective for percutaneous absorption [2]. However, the stratum corneum softening takes a long time, with side effects including redness, swelling, rash, and subjective feeling of burning sensation was caused in some users. Additionally, it may lead to hair growth on the undesired local with inaccurate using [3].

As a new type of painless administration, microneedles (MNs) with the advantages of convenient use, minimally invasive procedure, and relatively inexpensive cost, are easy to overcome stratum corneum to facilitate pharmaceuticals entering epidermal as well as dermal layers [4]. MNs are applied in many kinds of researches nowadays. For example, Li et al., last year, designed biodegradable MNs with an air bubble inside the center for rapidly separating chemicals to skin and caused sustained release [5]. However, the conventional fabrications of MNs are rigid, expensive, and inconvenient by directly purchasing commercial products, and using laser ablation, photolithograph, and magneto liquid forming, which prodigiously limits the applications of MNs [6,7,8,9,10,11,12].

Magnetic mesoporous nanoparticles consisted of silica as well as iron oxide, and are broadly employed in many fields such as catalyst, cancer therapy, antibacterial composites, energy storage, and drug delivery system [13,14,15,16,17,18,19]. These nano-systems provide promising and novel approaches for improving the efficacy with their large pore surface, which can also be easily controlled by adjusting the composition and concentration of surfactant. Especially, iron oxide nanoparticles allow them to be manipulated with externally applied magnetic field, which possess on-demand release behaviors from their cargos without any lag in response. By means of employing magnetic field, iron oxide can slightly generate heat to cause cutaneous blood flow increases as well as vasodilation, leading to the promotion of hair growth [20,21,22]. Furthermore, iron is one of the most important nutritional factors in human body, as it can help oxygen delivery as well as carbon dioxide removal from animal tissue, and has the composition of serum ferritin [23]. Many studies also show that iron-deficiency is also common with hair loss [24,25].

**Figure 1:**
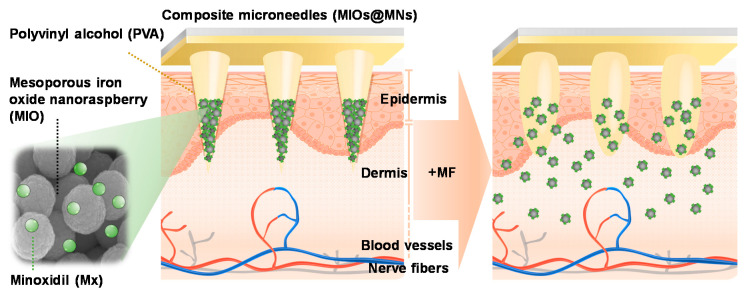
Schematic mechanism of microneedle patch system applying on the surface of skin. Polyvinyl alcohol (PVA) was used to prepare microneedles (MNs) with dissolvable properties. A mesoporous iron oxide nanoraspberry (MIO) encapsulating Minoxdil (Mx) inside the MNs were triggered by external magnetic field (MF), leading to local temperature increases as well as controlled release.

In this study, the concept of a physical promotion method for patching increases the efficiency of percutaneous absorption and reduces the chance of inducing side effects. A highly biocompatible polyvinyl alcohol was used to make a dissolvable microneedle patch, reaching the smart device in biomedical applications. Digital light processing (DLP) 3D printing technology was used to fabricate the MNs master with high resolution, inexpensive cost, flexible manufacturing, customization, and fast forming prototype compared with conventional process [26]. A mesoporous iron oxide nanoraspberry (MIO) inside MNs (MIOs@MNs) encapsulated Minoxdil (Mx), a therapeutic drug for hair regrowth, which can be triggered by external magnetic field (MF), resulting in local temperature increases as well as controlled release (Figure 1). We further evaluated the biocompatibility and mechanical properties of the MNs, suggesting a potential application in biomedical engineering filed. 

## 2. Materials and Methods 

### 2.1. Materials

FeCl_3_·6H_2_O, ethylene glycol, sodium acetate (NaAc), oleylamine, Mx, and phosphate buffered saline were purchased from Sigma-Aldrich, St. Louis, Missouri, USA. CdSe/ZnS core/shell Quantum Dot Solid (QDs) were purchased form Ocean Nano Tech, San Diego, California, USA. 3-(4,5-dimethylthiazol-2-yl)-2,5-diphenyltetrazolium bromide (MTT reagent) were bought from Amresco, Solon, Ohio, USA. Ham’s F-12 medium, fetal bovine serum (FBS) and penicillin/streptomycin were purchased from Gibco, Thermo Fisher Scientific, Waltham, Massachusetts, USA. Trypsin (2.21 mM EDTA Solution) was purchased from Corning, Steuben County, New York, USA. C57BL/6 mice obtained from National Laboratory Animal Center, NLAC, Taiwan.

### 2.2. Synthesis of MIOs

The synthesis process of MIOs follows the hydrothermal method of our previous studies [27]. Briefly, 5.4 g of FeCl_3_·6H_2_O were dissolved in 40 mL of ethylene glycol with vigorous stirring till the color of the solution changed to a clear yellow. Then, anhydrous NaAc (3.29 g) was added to the mixture in gentle stirring thereafter to the extent of homogeneity overnight. After the color of the mixtures changed to black, 0.6 mL of oleylamine was added under ultrasonication for effective mixing. Subsequently, 20 mL of the solution was transferred into a teflon-lined stainless steel autoclave (25 mL capacity) for hydrothermal reactions at 220 °C for 5, 15, and 25 h, respectively. After the Teflon-lined stainless steel autoclave was allowed to cool down to room temperature, the precipitate was washed by ethanol, three times, and stored in 4 °C, this was called MIOs. MIOs were dried under vacuum at room temperature before characterization and applications later. For Mx encapsulation, Mx was loaded to MIOs-5h by mixing 1% Mx with MIOs in dimethyl sulfoxide (DMSO) for 12 h. Then, the mixture was placed into vacuum at 50 °C for 24 h to evaporate DMSO. The drug concentration was determined by ultraviolet–visible (UV-vis) spectroscopy (Metertech, Taipei, Taiwan).

### 2.3. Fabrication Process of MNs

We used 3D printing technologies to prepare MNs master so that we can freely adjust the specification of MNs including height, width, and length of MNs. Each needle was 300 µm in a round base diameter and 600 µm in height. Needles were arranged with 600 µm tip-tip spacing. Polydimethylsiloxane (PDMS) was placed over the 3D printed MNs master to make female mold, followed by curing in the oven at 60 °C for 8 h, and the female mold was finally finished by carefully peeling the 3D printed MNs. For preparation polyvinyl alcohol (PVA)-MNs patch, the mixture, PVA and Mx-MIOs, were loaded onto PDMS female mold, and placed in a centrifuge at 4500 rpm for 1 h, so that the mixed solution would fill the pyramidal cavity of the mold. The back of MNs was filled with pure PVA solution, and centrifuged at 4500 rpm and 25 °C for 1 h. Subsequently, the device was allowed to dry at room temperature for 12 h, and then the patch was carefully detached from PDMS mold.

### 2.4. Characterizations of MIOs and MNs

The morphology of MIOs are analyzed by field emission scanning electron microscope (JEOL Ltd., Tokyo, Japan), and MIOs were dried up on silicon wafers. The wafers were anchored to SEM specimen mounts using double-sided carbon tape, and sputter deposited with platinum in 10 mA for 120 s. An X-ray diffractometer (D8 Advance X-ray Diffractometer, Bruker, Billerica, Massachusetts, USA) was used for identification of crystal dimensions. A thermogravimetric analyzer (Seiko Instruments Inc., Tokyo, Japan) provided a proportion of each compositions based on differential melting temperature with physical and chemical properties. Dynamics laser scattering (DLS) analysis, by using a particle sizer (Nano-ZS, Malvern, Worcestershire, UK), determined the average size and size distribution of MIOs diluted with deionized water. Field-dependent magnetization curves were evaluated by a superconducting quantum interference device (San Diego, California, USA) from −10,000 to 10,000 Oe.

### 2.5. Controllable Properties of MIOs

For heat generation studies, MIOs-5h, MIOs-15h, and MIOs-25h were inserted to the center of the coils, and MIOs were sequentially conducted with a MF (President Honor Industries, Taipei, Taiwan) at a power of 3.2 kW and frequency with 1 MHz. The temperature of solution increased and was detected at various times.

Mx was used to evaluate the drug loading capacity and release properties of MIOs. Then, 1% Mx were mixed with MIOs in DMSO for 12 h, and then the mixture was placed into vacuum at 50 °C for 24 h to evaporate the DMSO. After the organic solvent was evaporated, the mixture was washed by DI water through being centrifuged twice. Mx was quantified by a UV–vis spectrometer with the wavelength in 230 nm. The loading capacity of Mx in MIOs were calculated by the equation: EE% = (A − B)/A × 100, where A is the total amount of Mx, and B is the amount of Mx remaining in the supernatant. To estimate the drug release profile, Mx containing MIOs were dispersed in phosphate buffered saline (PBS). The solution was centrifuged at 4000 rpm and taken at various times for detection. 

For magnetically triggered release, MF was applied to activate and induce heat of MIOs at a power of 3.2 kW and frequency with 1 MHz for 1 min at the beginning. Mx-MIOs were dispersed in water and placed into tubes for detection at various times.

### 2.6. In Vitro Experiments

HIG-82 was obtained from the American Type Culture Collection (ATCC), and were maintained in F12 medium containing with 10% (*v*/*v*) FBS and 1% (*v*/*v*) penicillin-streptomycin at 37 °C and in 5% CO_2_. The culture medium was replaced every three days with a fresh one. For biocompatibility, we followed “Biological evaluation of medical devices—Part 5: Tests for in vitro cytotoxicity” (ISO 10993-5) to investigate the MNs. Briefly, we extracted the substance released from MNs in culture medium with serum following ISO 10993-12. After seeding HIG-82 cells in 96-well (10,000 cells per well) with 100 μL of medium for 24 h, we added the extraction into each well for another 24 h incubation. Finally, each well was added with 100 μL of MTT agent (1 mg/mL) for 4 h, and then dissolved in DMSO to make purple color liquid. The absorbance value was read with a microplate reader (Synergy™ HT Multi-detection microplate reader, BioTek Instruments, Inc., USA) with 570 nm in wavelength and 650 nm as reference. Cell viability was calculated by comparison with untreated cells and calculated according to the following: Cell viability (%) = absorbance of experimental group/absorbance of control group. 

### 2.7. In Vivo Experiments

All C57BL/6 mice aged 6 weeks were purchase from National Laboratory Animal Center, NLAC, Taiwan as the animal model of hair growth experiment. All mice were maintained under conditions at 22 ± 2 °C on a 12 h of dark-day cycle with access water and food. All surgical procedures were performed in accordance with the protocol approved by the Institutional Animal Care and Use Committee (IACUC), National Tsing Hua University, Hsinchu, Taiwan (IACUC protocol and approval number is 10704). Mice with 8 weeks of age were shaved and divided into 4 groups, including drug-containing control group (PBS), Mx-loaded MNs (Mx@MNs), Mx-MIOs@MNs, and Mx-MIOs@MNs+MF. Mice was treated with patches at day one after shaving. For control group, the shaved back of mice was covered with PBS solution. For Mx@MNs, Mx-MIOs@MNs, and Mx-MIOs@MNs+MF, the patch was pressed firmly for the first 30 s to penetrate through the epidermis and pressed softly for additional 2 min. The patch base was peeled at 10 min postinsertion into the skin, leaving the MNs settled in the skin for further sustained drug release. For Mx-MIOs@MNs+MF group, the mice were further treated under MF (power cube 32/900, President Honor Industries, Taiwan) at a power of 3.2 kW and frequency with 1 MHz. Photos of the mice back were recorded for the quantitation of the hair density by Zen Desk software. 

### 2.8. Statistical Analysis

As an analysis of the variance, both one- and two-way ANOVA were used, and statistical divergences were assessed by ANOVA with Tukey’s multiple comparison test. The Graph Pad-Prism was used with a significance level of alpha 0.05 to evaluate the statistical significance.

## 3. Results and Discussions

### 3.1. Synthesis and Characterization of MIOs

**Figure 2:**
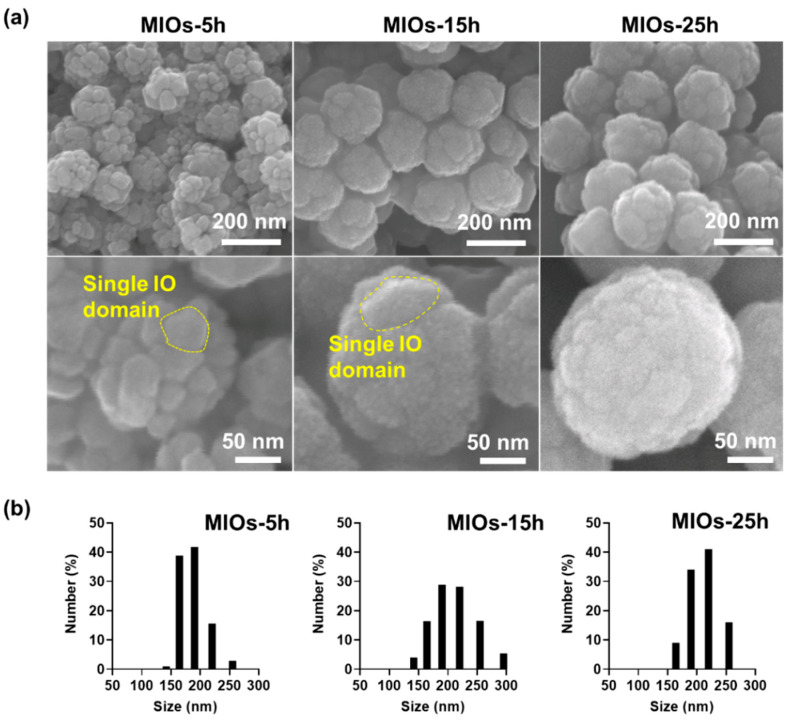
Morphologies and size distribution of MIOs. (**a**) scanning electron microscopy (SEM) images of different synthesis time (5 h, 15 h, and 25 h) of MIOs. (**b**) the size distribution of MIOs with different synthesis time (5 h, 15 h, and 25 h) measured by dynamic light scattering (DLS).

The synthesized process of MIOs was illustrated in Figure S1a. MIOs (porous iron oxide particles) were prepared by using ligand-aided synthetic approach, in which oleic amine (OA) and sodium citrate as coordinating agents was applied in a hydrothermal reaction [27]. Several methods, such as thermal decomposition, coprecipitation, or electrochemical, were able to fabricate the iron oxide based particles, the ligand-aided synthetic iron oxide that can manipulate the surface property, and the crystal growth rate to form the porous structures in one-step [28]. The magnetic particles serving as a drug delivery system are promising for on-demand drug release at a specific place since the external magnetic field was able to manipulate them remotely for spatial guiding and magneto-thermal conversion by a unique alternating MF [29]. After various reaction times, scanning electron microscopy (SEM) was applied to evaluate these MIOs as shown in Figure 2a. Based on the reaction time, the MIOs hereafter were termed as MIOs-5h, MIOs-15h, and MIOs-25h. Once the reaction was 5 h, the uniform MIOs-5h with clear pores could be observed, and it had a mean size of ~150 nm in diameter. At a closer observation (lower panel of Figure 2a), the MIOs-5h were composed by several iron oxide domains with 20 nm and the pores were constructed by these IO domains. Furthermore, iron oxide domains of MIOs were also observed by TEM (Figure S1b). 

The size and surface roughness of MIOs were affected by the reaction time. With reaction time ranged from 5 to 25 h, an increase of particle sizes was monitored from 150 to 200 nm, respectively. Moreover, the IO domains were also grown with time, causing the lower pore size and smoother surface for MIOs-15h and MIOs-25h. Similar size distribution was also detected by dynamic light scattering (DLS) as shown in Figure 2b. The plausible mechanism of fabrication for hydrothermal reaction has also been documented as an oriented gather and subsequent local Ostwald ripening [30], where the nucleation and growth of iron salts in solvent via the oriented growth of iron crystal and OA restricted the growth of crystal to form the pores. Furthermore, to evaluate the surface area, the Brunauer–Emmett–Teller (BET) method was applied to measure gas absorption isotherms (N_2_) at 77 K. The particles were treated at 80 °C under vacuum to remove the surface adsorption and were degassed at 180 °C for 4 h before BET analysis. The results revealed that each MIO-5h and MIO-10h had a surface area of ~265 and ~78 m^2^g^–1^, respectively (Figure S1c). Compared to MIO-5h, the lower surface area of MIO-10h was potentially attributed by the longer time of growth of iron oxide crystals. Moreover, such a OA ligand also adjusted the surface characteristics, efficiently improving the loading capacity of hydrophobic pharmaceuticals [30].

**Figure 3:**
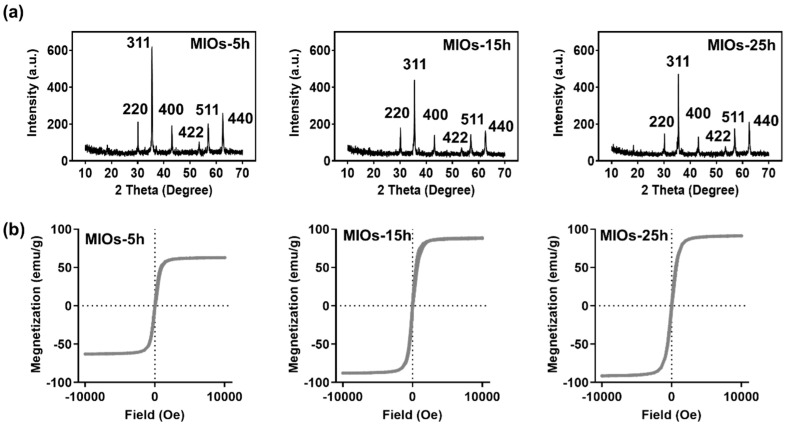
Characterization of MIOs. (**a**) X-ray diffraction (XRD). (**b**) superconducting quantum interference device (SQUID).

In Figure 3a, X-ray diffraction (XRD) analyses of MIOs exhibited the major diffraction planes at (200), (311), (400), (422), (511), and (440), which are the characteristic of the Fe_3_O_4_ crystal planes [31]. OA with primary amine, can adsorb onto building blocks as a coating agent, and also like a steric hindrance agent as a stabilizer during a hydrothermal reaction [32]. MIOs with different reactions represented identical characteristic peaks, and MIOs-5h has the smaller half width at (311) plane than MIOs-15h and MIOs-25h, suggesting the smaller grain size of IO particles. The results were also consistent to SEM images. Furthermore, a SQUID was utilized to investigate the magnetic properties of MIOs (Figure 3b). MIOs-5h, MIOs-15h, and MIOs-25h had similar magnetization-field patterns with negligible hysteresis but different magnetization saturations (Ms). The Ms of MIOs-5h was approximately 62 emu/g lower than MIOs-15h and MIOs-25h. As expected, while increasing the size of MIOs, an enhancing in Ms was observed. The difference was also reflected in the particle and grain sizes [33]. 

**Figure 4:**
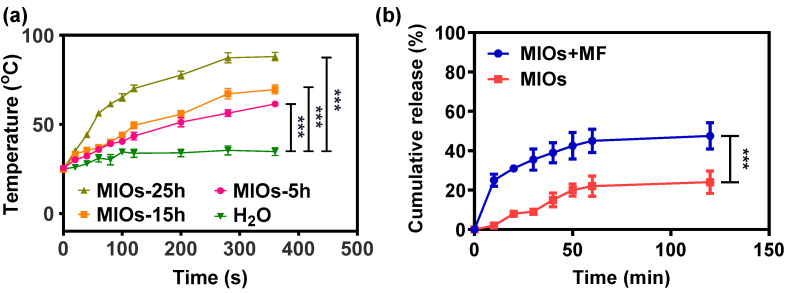
Controllable properties of MIOs. (**a**) temperature changes of MIOs solution under MF at a power of 3.2 kW and a frequency of 1 MHz. (**b**) cumulative release profile of Mx from MIOs with and without external MF treatment. Data points represent mean ± SD (N = 3, *** *p* < 0.005).

While 10 μg/mL of MIOs were subjected to a high frequency MF at a strength of 3.2 kW with a frequency of 1 MHz, an obvious temperature increase could be monitored (Figure 4a). Within 6 min of MF treatment, the temperature of solution with MIOs-25h increased to 86 °C. The mechanism of MF generating heat through magnetic matters can be understood by the energy relaxation and dissipation, known as Brown and Néel relaxations [34,35]. Brownian relaxation is due to rotational diffusion of the whole particle in the carrier liquid, and Néel relaxation is caused by the reorientation of the magnetization vector inside the magnetic core against an energy barrier. The energy was induced by the friction between particle–particle and particles–solvent under the alternating magnetic moments. On the other hand, the different IO domains with distinctive magnetic direction in one particle can also cause intense internal energy through the energy relaxation and dissipation. Therefore, both particle sizes and domains with various magnetic property affected the heating rates. After 6 min of MF application, the temperature of MIOs-5h was heated to 61 °C. The heating rate was affected by the size of iron oxide domain. The smaller iron oxide crystal usually offered the lower heating rate due to the weaker saturation of magnetization [36,37,38]. Therefore, MIOs-5h possessing smaller IO crystal sizes had a weaker heating rate in solution under MF treatment. For superparamagnetic iron oxide nanoparticles, the most influential analytic solution was derived using a linear response theory. The suitable magneto–thermal conversion was convenient for remotely controlled drug release and tissue treatment [39]. 

Considering the porosity and mild heating rate, MIOs-5h was used in the further studies. Figure 4b displayed the drug release profiles of MIOs-5h under MF. Before the release experiments, Mx was loaded to MIOs-5h by mixing 1% Mx with MIOs in DMSO for 12 h. Then, the mixture was placed into a vacuum at 50 °C for 24 h to evaporate DMSO. Estimated by the un-loaded Mx in supernatant, the loading efficiency of MIOs was about 88%, where loading was potentially attributed by the affinity between Mx and the large hydrophobic surface of MIOs-5h [40]. In the drug loading process of porous particles, oleic acid on the surface was usually utilized to offer hydrophobic–hydrophobic interaction for the physical adsorption of guest agents [30]. While coating hydrophobic ligands on the pore structure, the drug delivery system would efficiently improve the loading capacity of hydrophobic agents [40]. In the Mx loading, it was dissolved in organic solvent (DMSO) and mixed with MIOs. During the evaporation process for drug loading, the capillary motion of Mx would also potentially improve the loading efficiency. 

The Mx release from MIOs was monitored in PBS at 37 °C for 120 min (Figure 4b). Without treated by MF, a sustained released profile was detected, in which about 20% of Mx was released after 120 min. The slow release also reflected the affinity between Mx and MIOs. Once applying 1 min of MF to MIOs at the beginning, a faster release of Mx was monitored, and the treatment caused about 23% of the Mx to release within 10 min. Even the MF was turned off, the release was sustained. For 120 min, more than 40% of Mx can be released. The release was driven by the heat energy generated by MIOs, which was able to increase the molecules kinetics to overcome the adsorption on MIOs. Overall, the results indicated the on-demand Mx release from MIOs can be achieved by a MF. 

### 3.2. Fabrication and Characterization of MNs

**Figure 5:**
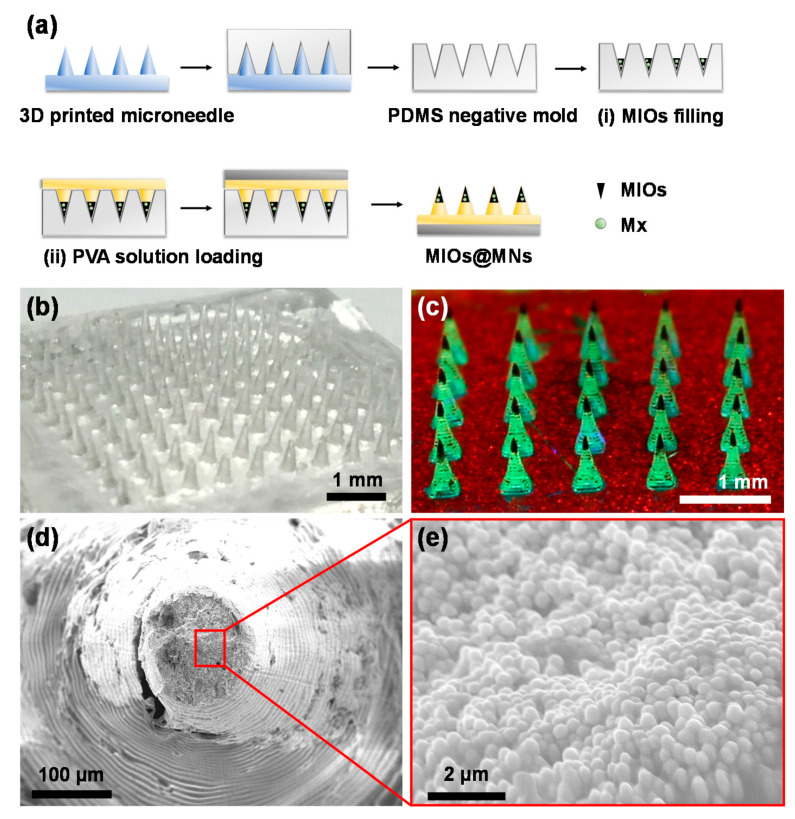
(**a**) Synthesis process of composite MNs with digital light processing (DLP) 3D printing and polydimethylsiloxane (PDMS) molding. Two steps of filling were carried out: (i) MIOs and (ii) PVA filling. The images of (**b**) PVA MNs and (**c**) composite MNs after mold preparation. MNs with MIOs (at needle tips) and green fluorescence PVA. SEM image of composite MNs in (**d**) low and (**e**) high magnification.

To prepare the MNs, PDMS served as the negative mold was reprinted according to the microneedle template printed in advance by 3D printing (Figure 5a). Due to the convenience of 3D printing, the different sizes and shapes of MNs can be designed and printed by digital light processing (Figures S2 and S3). In this study, for androgenetic alopecia treatment, the height of needle was designed as 600 μm with 11 by 11 array to reach the hair follicles. Using PDMS as a mold, 10 wt% of PVA with a molecular weight of 10,000 g/mol was filled, centrifuged, and dried in the mode. After solvent completely evaporated, the PVA MNs can be formed and separated from mold. The resulting PVA MNs was shown in Figure 5b. It provided the sharp and clear needle shapes with strong mechanical property. 

To form the composite MNs, a two step filling process was applied. First, the Mx-loaded MIOs (Mx-MIOs) were filled to PDMS modes. Followed by centrifugation and drying, the similar processes of PVA MNs were used to construct the MNs in the second step. The resulting composite MNs was shown in Figure 5c, in which MIOs as dark parts in MNs were observed in each tip. Once the green fluorescence dye was dissolved in PVA, for second layer in advance, the resulting fluorescence MNs could also be exhibited. The third layer labeled by red fluorescence could also easily formed. The results suggested that each layers of MNs can be established through the reliable step by step filling processes. Under SEM observation, the high resolution of composite MNs was investigated (Figure 5d). The ring-like patterns of MNs was caused by the DLS printing building the structures layer by layer. In the tip of MNs, the high density of MIOs was displayed, consistent to the previous results (Figure 5e).

**Figure 6:**
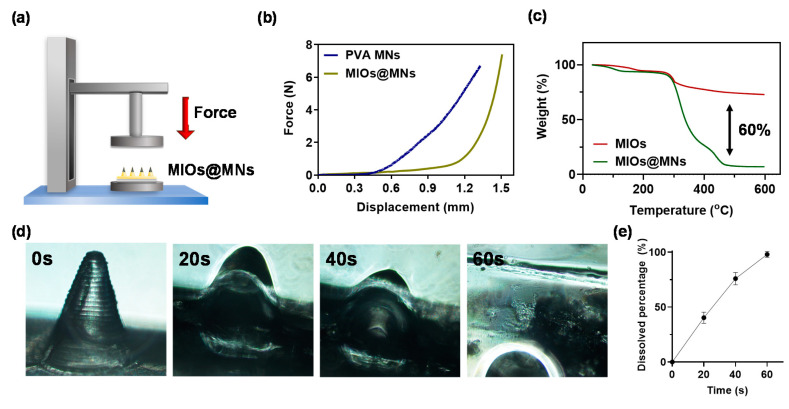
(**a**) schematic illustration of mechanical strength test for PVA and composite MNs. (**b**) compression displacement curve of PVA and composite MNs. (**c**) thermogravimetric analyzer (TGA) analysis of PVA and composite MNs. (**d**) optical images of dissolvable composite MNs in water at various times. (**e**) quantitation of dissolving rate.

Have demonstrated the structures of composite MNs, the mechanical property of composite MNs was estimated to confirm the ability to break through the stratum corneum barrier. The setup of the experiment was illustrated in Figure 6a, and the pressure versus displacement map obtained by applying pressure under the CT3 Texture (Figure 6b). The composite MNs were able to break the stratum corneum, where the minimum pressure required to break the stratum corneum was 0.17 N/needle [41]. Both PVA and composite MNs had sufficient mechanical strength to break the stratum. Furthermore, the thermogravimetric analyzer (TGA) was used to determine the weight ratio of MIOs and PVA in a single needle (Figure 6c). The result revealed the MIOs contributed 10% weight ratio in composite MNs, and 60% weight ratio of composite MNs was offered from organic materials such as PVA. 

In addition, we performed an in vitro microneedle swelling test to simulate the in vivo condition. PVA MNs swelled quickly within a minute (Figure 6d,e). This microneedle patch was proved to be soluble within a few seconds in water, and the fast dissolution characteristics have great potential for the delivery of the drug, followed by decomposing the patch through skin interstitial fluid. The experimental results show that the microneedle patch can effectively damage the stratum corneum and dissolve in the skin, and the transmission efficiency should be improved for the drug in the transdermal route.

### 3.3. In Vitro Experiments

The MNs were applied to a pork skin to investigate the insertion (Figure S4a). With 10 min of MF applications, the skin surface was cleaned to remove the excess MNs. Under the images, the clear MNs patterns can be detected on the skin surfaces, indicating the MN was able to insert into the skin. The histological evaluation also further confirmed the MNs insertion (Figure S4b), in which the barrier of the stratum corneum was broken. 

**Figure 7:**
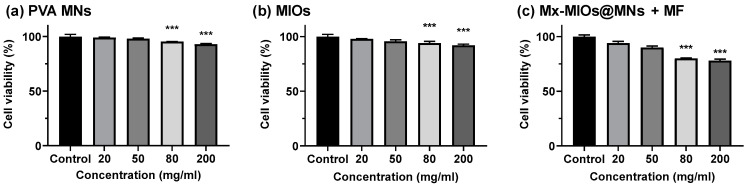
The cell viability of HIG-82 cells incubated with (**a**) PVA MNs, (**b**) MIOs, and (**c**) Mx-MIOs MNs+MF. Data points represent mean ± SD (N = 5, *** *p* < 0.005).

Having exhibited the insertion of MNs, the toxicity of PVA and MIOs@MNs were evaluated in Figure 7. The in vitro cell compatibility of MNs were determined using fibroblast cells (HIG-82). Briefly, HIG-82 cells were plated on 96 well microplates at a density of 1 × 10^4^ cells/mL. Then, the culture medium was changed, and cells were exposed to serial dilutions of tested dissolved MNs. Cell viability was assessed after 72 h by means of MTT assay by measuring absorbance at 570 nm wavelength using ELISA Reader. In Figure 7a, the cell viability decreased with increasing PVA concentrations, but the cell viability can measure more than 90%, suggesting the low toxicity of PVA MNs. The cell viability of MIOs in Figure 7b also performed similar behaviors and weak influences in cell viability even at the Mx-MIOs@MNs at concentration of 200 mg/mL. While applying 1 min of MF, the higher cell toxicity was observed in composite MNs. The cell death was potentially caused by the heat. Although the temperature was less than 40 °C with 1 min treatment, the local energy may affect the cell proliferation at high concentration. However, the cell viability can still keep at 75% at the concentration of 200 mg/mL.

### 3.4. In Vivo Animal Hair Growth Experiments

**Figure 8:**
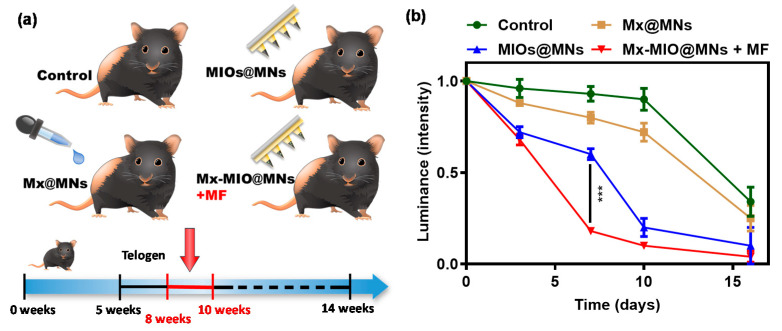
In vivo evaluation of dissolvable MNs for hair loss treatment. (**a**) schematic illustration represents control, Mx@MNs, Mx-MIOs@MNs, and Mx-MIOs@MNs+MF groups for hair loss treatment for 16 days. (**b**) the hair intensity treated by control, Mx@MNs, Mx-MIOs@MNs, and Mx-MIOs@MNs+MF groups for hair growth on mice. Data points represent mean ± SD (N = 3, *** *p* < 0.005).

**Figure 9:**
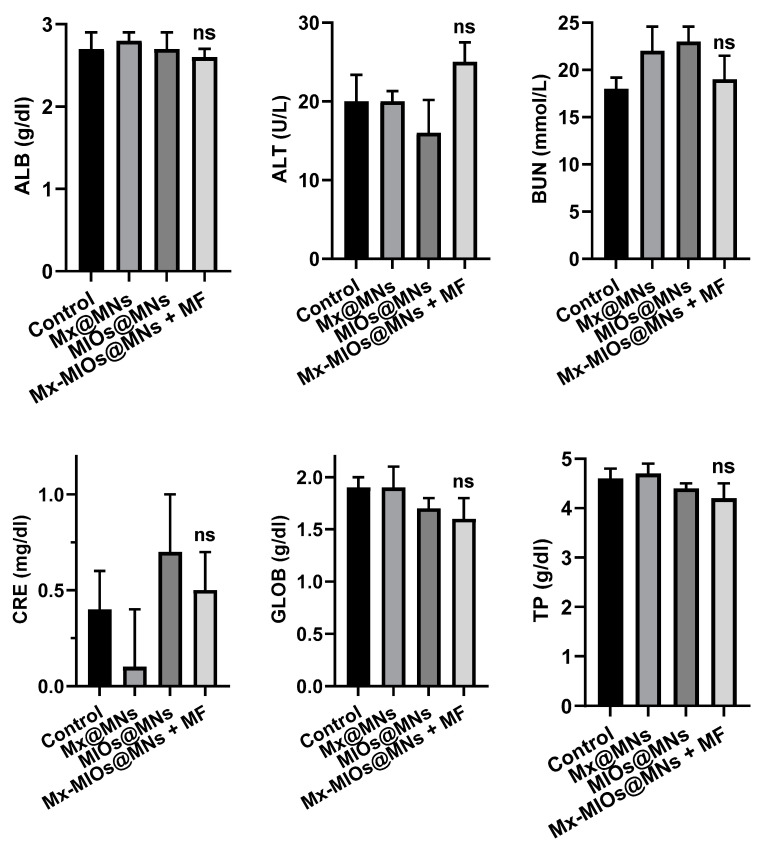
The toxicity of liver and kidney. Kidney functions of albumin (ALB), alanine aminotransferase (ALT), blood urea nitrogen (BUN), creatinine (CRE), globulin (GLOB), and total protein (TP) of mice treated by Mx@MNs, Mx-MIOs@MNs, and Mx-MIOs@MNs+MF (n = 3, mean ± SD, ns: No significant difference).

The in vivo animal hair growth studies were carried by 8 weeks-old C57BL/6 mice. Before treating by MNs, the hairs were removed by shaving and hair removal cream. Then, C57BL/6 mice were divided into four groups, including control, Mx-loaded MNs (Mx@MNs), Mx-MIOs@MNs, and Mx-MIOs@MNs+MF (Figure 8a). A microneedle patch (11 by 11) prepared by the above-mentioned experimental optimization formula was administered at the 8th week, and the administration area was 1 cm^2^. The hair was applied to the back of the hairless mouse and peeled off after 10 min of attachment. The same dosage of Mx was used in the four groups. After photographing the back of the mouse, a fixed range was tracked for 16 days and taken to quantify the percentage of the selected range of hair by the computer software ZEN zeiss at various times (Figure S5). The results of hair growth were exhibited in Figure 8b. At the first time point, the bright intensity was normalized as baseline. At the 3rd and 7th days of treatment, the obvious difference could be observed, where the mice treated by Mx-MIOs@MNs demonstrated 25% and 40% of hair re-growth, respectively, while no hair was found regrowth beyond the treated region. However, in the sharp contrast, the control group generated an inferior therapeutic effect, showing less than 12% recovery of hair within 1 week. No obvious hair regrowth was found in the mouse without simultaneous MF treatment and Mx during the test period, indicating that the hair follicles were still in the telogen phase of the hair cycle [42]. Once applying MF to Mx-MIOs@MNs, the faster hair growth rate can be monitored within two weeks. At the 10th day, the hair density of Mx-MIOs@MNs+MF was eight-fold greater than that of control group, indicating the improvement of thermal-derived Mx release and penetration. Furthermore, both liver and kidney functions were evaluated to understand the toxicity of treatments. Six indices of liver and kidney functions including albumin (ALB), alanine aminotransferase (ALT), blood urea nitrogen (BUN), creatinine (CRE), globulin (GLOB), and total protein (TP) were also evaluated at 3-day posttreatment (Figure 9). The results revealed that the PVA and composite MNs with MF treatment caused negligible toxicity.

## 4. Conclusions

In summary, magneto-responsive transdermal composite microneedles with a mesoporous iron oxide nanoraspberry were developed to improve androgenetic alopecia (AGA) treatment. Unlike traditional administration, this approach can reduce side effect from treating on the undesired local with inaccurate use. MIOs with porous structure and hydrophobic surface can capture hydrophobic pharmaceuticals, which also are the most important nutrition avoiding hair loss in human body. Besides, under the non-contact trigger, MF, MIOs, would generate wild heating for aiding increases of cutaneous blood flow as well as vasodilation. Minoxidil is a drug for hair regrowth with high efficiency. By using 3D printed technology, it can build various morphologies microneedle molds for further uses according to the geometry of tissue anatomies. Through filling Mx-loaded MIOs and PVA subsequently to mold to MNs, the layer-by-layer composite MNs is constructed with high mechanical property for overcoming the barrier of the stratum corneum via the punctuation, and dissolved, later, after administration. The successful delivering the Mx-loaded MIOs to skin and integrating the magneto–thermal drug release achieved eight-fold greater improvements of hair growth with low toxicity. This composite MNs provides a new synergistic delivery strategy for transdermal drug delivery and potential for wide clinical applications.

## Supplementary Materials

The following are available online at https://www.mdpi.com/2073-4360/12/6/1392/s1, Figure S1: Synthesis process of MIOs and hydrothermal reaction; Figure S2: Schematic of fabrication of MNs by 3D printing; Figure S3: Image of microneedle surface puncture; Figure S4: Skin insertion of MNs; Figure S5: Comparison in hair growth in an alopecia model of C57BL/6 mice applied with test compound topically for over two weeks.
