# Supplementary material for: Transdermal Composite Microneedle Composed of Mesoporous Iron Oxide Nanoraspberry and PVA for Androgenetic Alopecia Treatment
Source: Polymers (Basel). 2020 Jun 22;12(6):1392. doi: 10.3390/polym12061392 (PMC7362218; doi:10.3390/polym12061392)
Supplement: Supplementary file 1 [file polymers-12-01392-s001.pdf]

Supplementary

Transdermal Composite Microneedle Composed of Mesoporous Iron Oxide Nanoraspberry and PVA for Androgenetic Alopecia Treatment

Jen-Hung Fang [1], Che-Hau Liu [1], Ru-Siou Hsu [1], Yin-Yu Chen [1], Wen-Hsuan Chiang [2], Hui-Min David Wang [3] and Shang-Hsiu Hu [1,*]

[1] Department of Biomedical Engineering and Environmental Sciences, National Tsing Hua University, Hsinchu 300, Taiwan; s102012803@m102.nthu.edu.tw (J.-H.F.); u9912052@gms.ndhu.edu.tw (C.-H.L.); hsu.ru.siou@gmail.com (R.-S.H.); anny99943@gmail.com (Y.-Y.C.)

[2] Department of Chemical Engineering, National Chung Hsing University, Taichung 402, Taiwan; whchiang@dragon.nchu.edu.tw

[3] Graduate Institute of Biomedical Engineering, National Chung Hsing University, Taichung 402, Taiwan; davidw@dagon.nchu.edu.tw

* Correspondence: shhu@mx.nthu.edu.tw

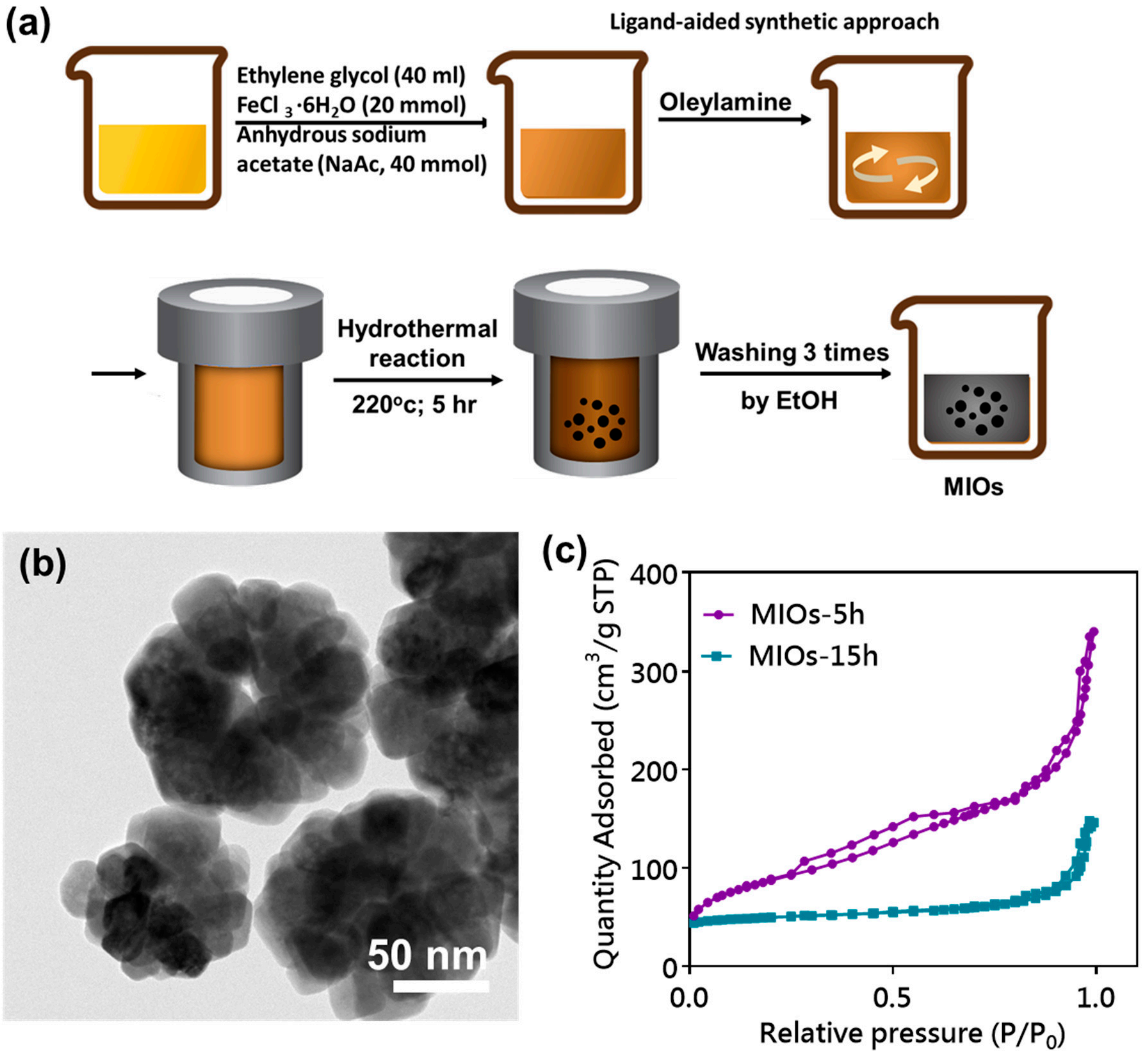

Figure S1. (**a**) Synthesis process of MIOs and hydrothermal posttreatment. (**b**) TEM images of MIOs-5h. (**c**) N_2 adsorption–desorption isotherms of MIOs-5h and MIOs-15h.

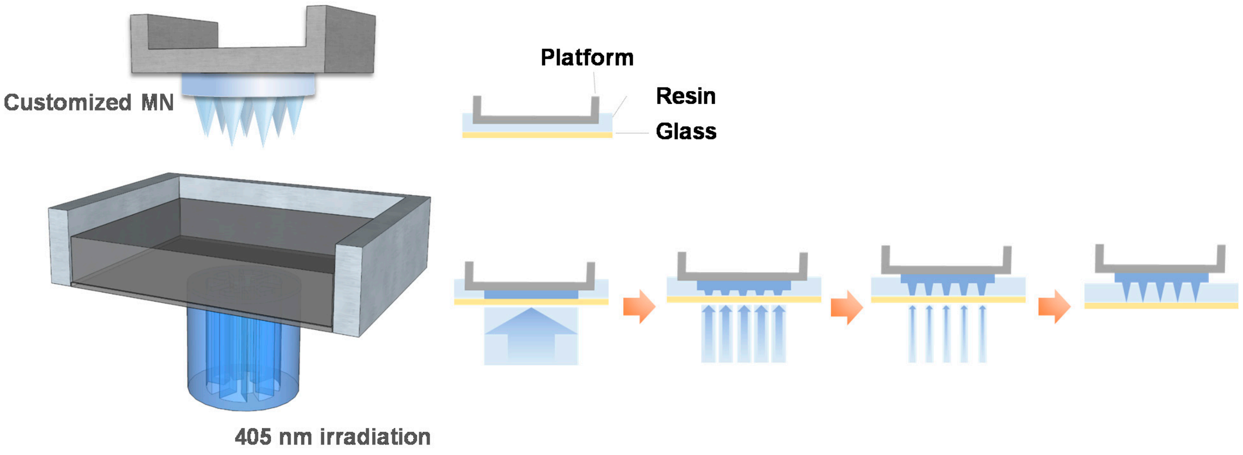

Figure S2. Schematic of fabrication of MNs by digital light processing (DLP) 3D printing.

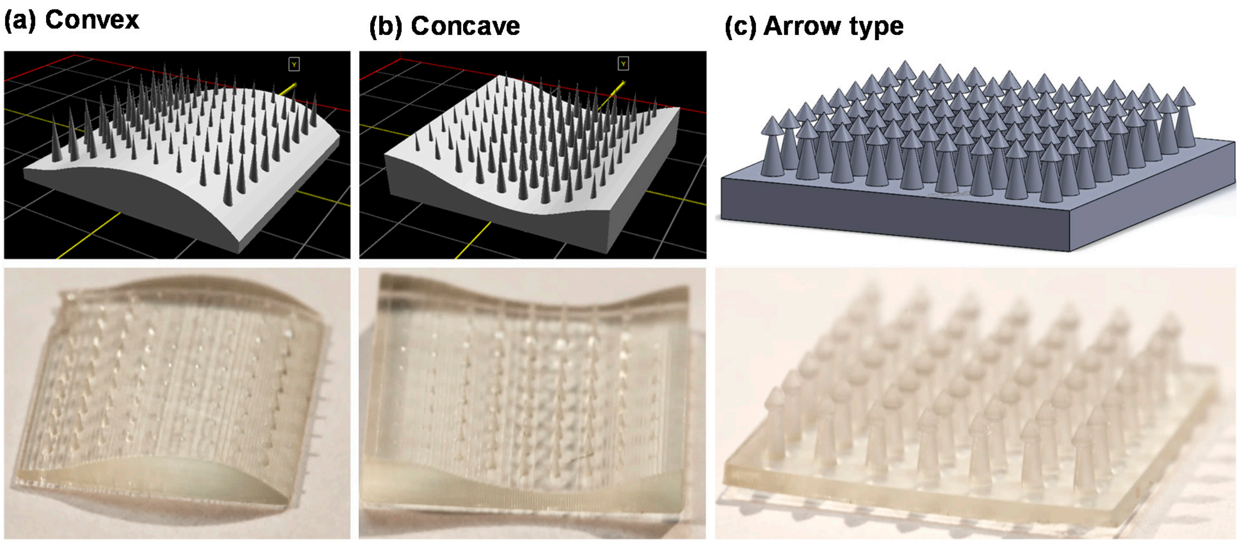

Figure S3. The different sizes and shapes of microneedles can be designed and printed by digital light processing (DLP) process. (**a**) Convex, (**b**) concave and (**c**) arrow type.

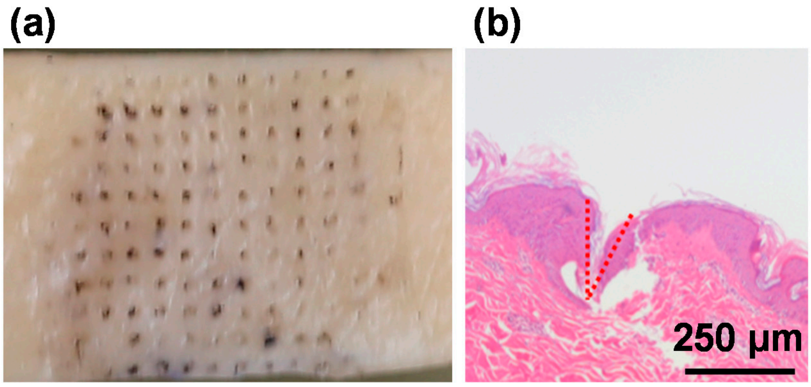

Figure S4. Skin insertion of MNs. (**a**) image of skin after insertion. (**b**) H&E stained cross-section.

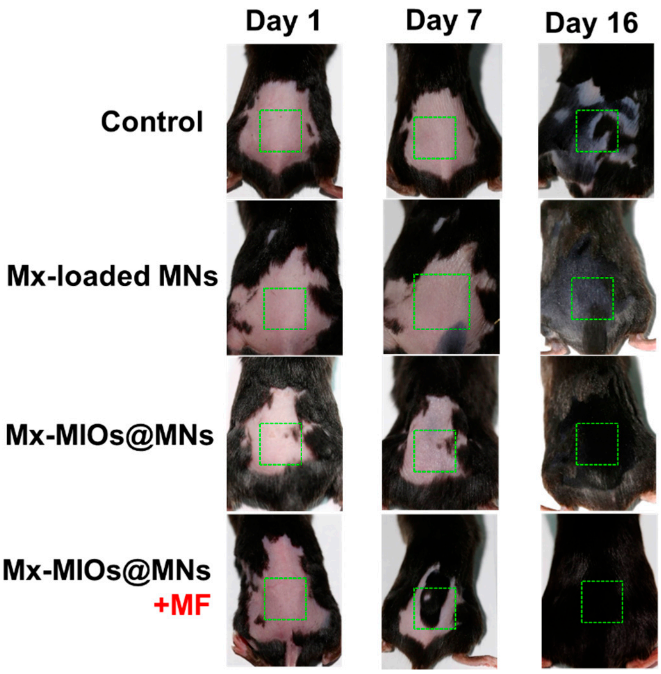

Figure S5. Comparison in hair growth in an alepocia model of C57BL/6 mice applied with test compound topically for over two weeks.
